# Effects of Synthetic Ligustrazine-Based Chalcone Derivatives on *Trypanosoma brucei brucei* and *Leishmania* spp. Promastigotes

**DOI:** 10.3390/molecules28124652

**Published:** 2023-06-08

**Authors:** Abdulsalam A. M. Alkhaldi

**Affiliations:** Biology Department, College of Science, Jouf University, Sakaka 72341, Saudi Arabia; abdulsalam@ju.edu.sa or a.slam97@hotmail.com

**Keywords:** trypanosomiasis, *Leishmania* spp., sleeping sickness, chalcones, ketones, toxicity, ligustrazine, protozoan parasites

## Abstract

Current medication therapy for leishmaniasis and trypanosomiasis remains a major challenge due to its limited efficacy, significant adverse effects, and inaccessibility. Consequently, locating affordable and effective medications is a pressing concern. Because of their easy-to-understand structure and high functionalization potential, chalcones are promising candidates for use as bioactive agents. Thirteen synthetic ligustrazine-containing chalcones were evaluated for their ability to inhibit the growth of leishmaniasis and trypanosomiasis in etiologic agents. The tetramethylpyrazine (TMP) analogue ligustrazine was chosen as the central moiety for the synthesis of these chalcone compounds. The most effective compound (EC_50_ = 2.59 µM) was the chalcone derivative **2c**, which featured a pyrazin-2-yl amino on the ketone ring and a methyl substitution. Multiple actions were observed for certain derivatives, including **1c**, **2a–c**, **4b**, and **5b**, against all strains tested. Eflornithine served as a positive control, and three ligustrazine-based chalcone derivatives, including **1c**, **2c**, and **4b**, had a higher relative potency. Compounds **1c** and **2c** are particularly efficacious; even more potent than the positive control, they are therefore promising candidates for the treatment of trypanosomiasis and leishmaniasis.

## 1. Introduction

There are a number of major neglected tropical diseases (NTDs), but three of the most important are leishmaniasis, human African trypanosomiasis (HAT) (also known as sleeping sickness), and American trypanosomiasis (also known as Chagas disease). In South America, leishmaniasis and Chagas disease are caused by similar trypanosomatid protozoan parasites, and in Africa, leishmaniasis and human African trypanosomiasis (HAT) are co-endemic [1,2,3]. Human and animal populations continue to be threatened by African trypanosomiasis. The lack of effective treatments, drug toxicity, and drug resistance have kept the disease at bay, making it imperative to learn more about the parasite’s biology and develop new treatments. HAT and animal African trypanosomiasis (AAT) are two forms of the same neglected tropical disease that pose serious risks to both human and animal populations in endemic areas [4,5]. Between 2016 and 2020, it was predicted that 55 million people in 36 countries across sub-Saharan Africa will be susceptible to HAT [6], which is fatal unless treated. From approximately 40,000 cases in 1998, there were only 992 and 663 new cases recorded in 2019 and 2020, respectively [6]. This represents a substantial drop in the number of cases that has been brought about by multiple control techniques. Even though the illness has not been completely eliminated, its prevalence has been drastically cut down [7]. Given that most patients live in rural areas and conflict zones, these numbers are likely to be low. However, African trypanosomiasis in animals is still a major problem in sub-Saharan Africa [8]. The annual mortality toll from this disease is estimated to be around 3 million, and it is responsible for a yearly economic loss in cow output of around USD 1.0–1.2 billion [7].

Cattle can contract nagana, also known as animal African trypanosomiasis, from the parasite *Trypanosoma brucei brucei*. Three million cattle are lost every year to the disease, which threatens about 55 million [9]. There is currently no vaccination available for trypanosomes due to the antigenic diversity displayed by the parasites; hence, chemotherapy is the major form of treatment [10]. Current drugs are dangerous because they are poisonous, have undesirable side effects, and are losing their efficacy as a result of drug resistance. As a result, there is an urgent demand for the research and development of new treatments for *Trypanosoma* that are not only efficient but also risk-free.

Leishmaniasis is an often fatal, parasitic protozoal illness spread mostly via the bite of an infected insect in warm, humid environments [11]. *Leishmania* spp., a genus of flagellated protozoa, contains over 20 species that are accountable for the illness [12]. The clinical manifestations of leishmaniasis can be divided into two categories. The cutaneous form of leishmaniasis (CL) is by far the most common form of the disease. Because of its catastrophic effects on the skin, which make it difficult for many patients to go about their normal activities, it has become a serious issue in international healthcare. *L. major* and *L. tropica* are the species that are responsible for the majority of CL cases across the globe. The presence of the sand fly *Phlebotomus papatasi* in regions where *L. major* infections are common is likely to blame for the vast majority of reported cases of the disease in Saudi Arabia and other Arabian countries [13]. The zoonotic form of CL evolved as a result of the global expansion of leishmaniasis in the 20th century, with an estimated yearly incidence in Saudi Arabia of more than 4000 [14,15]. The widespread prevalence of desert rodents (reservoir animals) and sand flies is largely responsible for the endemic nature of CL in several regions in Saudi Arabia. [16,17]. Recent research into the incidence of leishmaniasis in Central Saudi Arabia’s Qassim Province found that *L. major* accounts for 50% of cases, *L. tropica* 29%, and *L. infantum* 4% [12].

For the treatment of trypanosomiasis and leishmaniasis, only a handful of antiquated medications are available; unfortunately, these treatments all have similar low safety, effectiveness, and pharmacokinetic profiles. There has been no new clinical drug introduced in decades, despite public and private attempts to speed up the drug discovery process in recent years. In order to effectively combat these illnesses, new and better medications must be developed. These drugs should have high pharmacological activity and bioavailability, low toxicity, a suitable dosing regimen, and parasite resistance bypassing [18].

In light of our prior findings [19], I have gone above and beyond the scope of our previously reported research in an effort to identify novel compounds with antiprotozoal activities [20], which included ligustrazine (tetramethylpyrazine)-based chalcones with new pyrazin-2-yl amino and quinazolin-4-yl amino moieties. In traditional Chinese medicine, TMP is found in the chuanxiong plant (*Ligusticum chuanxiong* Hort). Based on their high antiparasitic efficacy and minimal toxicity, synthesized oxime and cyclohexanone compounds were identified in a prior investigation as having potential applications against trypanosomiasis and leishmaniasis. Because of their activity against both strains of trypanosomiasis, the compounds identified in that investigation provide promising leads for further structure-based optimization and development against *T. brucei brucei* [19]. Several strains of *Leishmania* spp. and *Trypanosoma* were tested in this investigation of novel α,β-unsaturated carbonyl-based compounds coupled to TMP moiety.

## 2. Results and Discussion

In the present study, I demonstrated the effect of synthetic compounds on the growth of promastigotes of *L. major, L. mexicana*, and a wild-type strain of *Trypanosoma brucei brucei* (s427-WT) in vitro. The antitrypanosomal and leishmanicidal activities of the compounds are summarized in Table 1.

### 2.1. Antitrypanosomal Activity of Tetramethylpyrazine Containing Chalcone Derivatives

In vitro trypanocidal activity against wild-type (WT) *Trypanosoma brucei brucei* was tested for all 13 synthetic α,β-unsaturated carbonyl-based tetramethylpyrazine containing chalcone derivatives (Table 1). Ten compounds inhibited the strain, and their EC_50_ values were quite high. The chalcone derivative **2c** showed the strongest inhibitory activity (EC_50_ = 2.59 µM), followed by the chalcone derivative **4b** with EC_50_ = 22.8 µM, which was nearly as active as **1c** with EC_50_ = 23.3 µM.

In this study, eflornithine and pentamidine served as positive controls, and it was found that three compounds were significantly more potent than the positive control eflornithine: **1c** exhibited the same activity as eflornithine, **2c** was ten times more potent, and **4b** was also more effective than the positive control. Despite eflornithine’s original development for oncology purposes, it was later found to be an efficient antitrypanosomal therapy. It is on the WHO’s proposed template for a roster of essential medicines.

All of these compounds feature a central α,β-unsaturated carbonyl-based linker that has been variously replaced with functional groups. Based on the functional moieties that are connected, these compounds fall into one of five categories. In the first category are some recently discovered chalcone derivatives based on ligustrazine that include a quinazolin-4-yl amino substitution in the ketone ring’s position 4 (**1a–c**). The second class consists of unheard-of pyrazin-2-yl amino which substituted ketone-4 ligustrazine-based chalcone derivatives (**2a–c**). The next three classes are simple chalcones; all of these compounds contain ligustrazine in their aldehyde moiety, while different classes of ketones on the other side of **3a–c** bear methyl-substituted ketones, **4a–b** bear 1-Pyridin-3-yl-ethanone as a ketone moiety, and lastly **5a–b** have pyrazine in ketone moiety.

There are three compounds in this first class of ligustrazine-based chalcone derivatives (**1a–c**): compound **1a**, which has no substitutions; compound **1b**, which has a chlorine at R_2_; and compound **1c**, which has trimethoxy replacements at R_2_, R_3_, and R_4_. By comparing the trypanocidal activities of the tested compounds (Table 1), it was found that the substitution pattern had a significant impact on the results, with compounds **1a** and **1b** containing no substitution and **1c** containing trimethoxy substitutions at R_2_, R_3_, and R_4_ demonstrating three times the potency with an EC_50_ = 23.3 µM.

The most interesting and potent effects were exhibited by the second type of ligustrazine-based chalcone derivatives with pyrazin-2-yl amino substitution at position 4 on the ketone ring (**2a–c**). Among these three compounds (**2a–c**)were one derivative **2a** without any substitution and one derivative **2b** in the group bearing methyl substitution at R_1_ position which exhibited the same trypanocidal activity, but the third derivative **2c** in the group bearing methyl substitution at R_2_ position on the pyrazin-2-yl amino moiety showed the highest inhibition as compared to all test compounds in this study with EC_50_ = 2.59 µM.

All other tested compounds were simple chalcone derivatives without any extension on ketone moiety as compared to **1a–c** and **2a–c**. Among these seven chalcone derivatives were three compounds (**3a–c**) bearing methyl-substituted ketones, two derivatives (**4a–b**) bearing 1-Pyridin-3-yl-ethanone as a ketone moiety, and two derivatives **5a–b** having pyrazine in ketone moiety which showed inhibition of *Trypanosoma brucei brucei* with EC_50_ ranging from 22.0 to above 100.0 µM. On the other hand, one derivative **4b** with 1-Pyridin-3-yl-ethanone was found to be a strong inhibitor for the tested strain.

Previously, we [19] found that cyclohexanone derivatives, with a tetramethylpyrazine moiety on both sides of the central cyclohexanone linker, were more active than the respective oximes against the protozoa of the *Trypanosoma* and *Leishmania* spp. species when tested in a series of 34 synthetic ligustrazine-containing, unsaturated carbonyl-based compounds and oximes [19]. Hicks and co-workers synthesized and characterized a series of chalcones with a prenyloxy or geranyloxy unit, and they discovered that the position of the substituents in the molecule, whether on the ketone or aldehyde ring of the chalcone system, affected the biological activity and the selectivity index of these compounds [21].

### 2.2. Antileishmanial Activity of Synthesized Chalcone Derivatives

The same 13 synthetic ligustrazine chalcone derivatives were tested on promastigotes of *L. major* and *L. mexicana* (Table 1). Only the first group containing ligustrazine-based chalcone derivatives with quinazolin-4-yl amino substitution at position 4 on the ketone ring (**1a–c**) and the second group containing ligustrazine-based chalcone derivatives with pyrazin-2-yl amino substitution at position 4 on the ketone ring (**2a–c**) showed inhibition of both *L. major*. and *L. mexicana* with EC_50_ values in the range 27.7–92.7 µM for *L. mexicana and* EC_50_ values in the range of 15.7–65.9 µM for *L. major* for **1c** and **2a–c**, while **1a–b** were found to be inactive for *L. major.*

The remaining three groups of chalcones with ligustrazine on the aldehyde portion; **3a–c** bearing methyl-substituted ketones, **4a–b** bearing 1-Pyridin-3-yl-ethanone as a ketone moiety, and lastly **5a–b** having pyrazine in ketone moiety; were found to be almost inactive or far less effective against both *L. mexicana* and *L. major*.

Twenty-five new prenylated chalcones [22] with antiparasitic efficacy against *Leishmania mexicana* were recently synthesized. The selectivity index (SI) values for these chalcones were also the highest. Antileishmanial action may be affected by the substitution pattern, as shown by the fact that two chalcone derivatives with a meta position substituent in the B ring have been found to exist. In addition, homology modeling was used to generate a three-dimensional structure of *L. mexicana* fumarate reductase. According to docking studies, prenylated chalcones have the potential to regulate fumarate reductase activity by binding to two essential binding sites [22].

## 3. Materials and Methods

### 3.1. Materials

Compounds of synthetic origin that were tested in this research were ones that we had previously synthesized, characterized, and reported on [20] (Table 2). Each compound was used to prepare a stock solution in 100% DMSO; afterward, the concentration of that standard solution was adjusted appropriately for the experiment by diluting it with complete medium, with the final DMSO content kept below 1%.

### 3.2. Cell Culture

#### 3.2.1. *Trypanosoma brucei brucei* s427-WT

*Trypanosoma brucei brucei* s427-WT, the wild-type strain, was employed. According to the method reported by Hirumi and Hirumi, the optimal conditions for the development of this strain were a pH 7.4 HMI-9 medium supplemented with 10% heat-inactivated fetal calf serum (FCS, BioSera) and 14 µL/L of 13.4 M -mercaptoethanol (Sigma-Aldrich, St. Louis, CA, USA) [23]. In a flow cabinet, the medium was filtered at 0.22 m (Millipore, Burlington, MA, USA) to remove any bacteria. *T. brucei brucei* was grown in an incubator and passed three times a week in vented flasks at 37 °C and 5% carbon dioxide.

#### 3.2.2. *Leishmania* spp. Promastigotes

In essential medium (HOMEM) at 25 °C, pH 7.4, and 10% FCS in polyethylene flasks, we cultivated *Leishmania major* Friedlin (LmjF) and *Leishmania mexicana* (MNYC/BZ/62/M379). All of the *Leishmania* spp. strains were grown as promastigotes in a standard HOMEM (GIBCO, Life Technologies, Paisley, UK) medium that was augmented with 10% heat-inactivated fetal bovine serum (FBS; PAA Laboratories, Linz, Austria) and 1% of a penicillin–streptomycin solution (Life Technologies, Carlsbad, CA, USA) and incubated at a temperature of 25 °C. The cultures were swapped out for new media three times a week.

### 3.3. Alamar Blue Assay

As an indication of metabolic function in cells, resazurin sodium salt (Alamar Blue) is widely utilized. To assess the in vitro susceptibility of African trypanosomes or *Leishmania* spp. cells to test substances, a nonfluorescent blue dye was added to cell cultures with different drugs doses [24,25]. In the absence of drug-induced toxicity, live cells glow a bright red instead of a dull blue. The Alamar Blue solution was made by dissolving 12.5 mg of resazurin sodium salt (Sigma) in 100 mL of phosphate-buffered saline (PBS) at pH 7.4, filter-sterilizing the solution, and then storing it in the dark at 4 °C.

### 3.4. Drug Sensitivity

Using a 20 mM stock solution in DMSO, a solution of 200 µM in HMI-9 medium + 10% FCS was made, and 200 μL of this was put into the first well of a 96-well plate for each test chemical. One hundred microliters of this solution was transferred to a well that already had one hundred microliters of the same medium in it, creating a 1:1 dilution and setting in motion a doubling dilution series across two rows of the plate. In all experiments involving *Trypanosoma brucei brucei*, eflornithine and pentamidine served as positive controls, whereas for *Leishmania* spp. promastigotes, only pentamidine served as a positive control, and HMI-9 medium was used as a negative, drug-free control in the final well for each compound. Each well received 100 μL of a 2 × 10^5^ cells/mL cell solution, for a final cell density of 1 × 10^5^ cells/mL. After 48 h incubation at 37 °C and 5% carbon dioxide, 20 μL of the Alamar Blue solution was added and the plate was put back in the incubator for another 24 h. The plate’s fluorescence was measured using a fluorimeter (FluoStar Optima) at excitation and emission wavelengths of 590 nm and 530 nm, respectively; the data were then analyzed using the GraphPad Prism 5 software package, and the EC_50_ value was calculated by fitting the fluorescence to a sigmoid curve with a variable slope. Using *Leishmania* spp. promastigotes, a very similar procedure was performed [26].

## 4. Conclusions

Large numbers of individuals in underdeveloped nations are afflicted by neglected tropical diseases including leishmaniasis and trypanosomiasis, both of which are prevalent in tropical locations. The antileishmanial and antitrypanosomal actions of a panel of 13 ligustrazine-based chalcones were tested in vitro. Some chalcones with quinazolin-4-yl amino substitution at position 4 on the ketone ring (**1c**) and with pyrazin-2-yl amino substitution at position 4 on the ketone ring (**2a–c**) displayed a strong growth inhibition of the promastigotes of *L. major, L. mexicana*, and a wild-type strain of *Trypanosoma brucei brucei* (s427-WT) in vitro. *Trypanosoma brucei brucei* growth suppression was enhanced by compounds **1c, 2c,** and **4b** compared to the positive control eflornithine. These findings provide evidence for the therapeutic potential of ligustrazine-based chalcone derivatives. Generally speaking, ligustrazine-based chalcone derivatives with pyrazin-2-yl amino and quinazolin-4-yl amino substituents may be promising for the creation of new therapeutic medications against parasite illnesses.

## Figures and Tables

**Table 1:** Effect of synthetic chalcone derivatives on *Trypanosoma brucei brucei*, *Leishmania major*, and *Leishmania mexicana* strains.

No.	Compounds	*Trypanosoma brucei brucei* EC_50_ µM	*Leishmania* spp. Promastigotes EC_50_ µM	
		(WT)	*L. major*	*L. mexicana*
1.	**1a**	65.24 ± 1.82	>100	92.70 ± 1.320
2.	**1b**	65.24 ± 1.82	>100	49.27 ± 3.893
3.	**1c**	23.32 ± 0.80	15.78 ± 2.29	36.41 ± 1.315
4.	**2a**	30.53 ± 1.10	65.97 ± 2.93	67.03 ± 2.823
5.	**2b**	30.59 ± 2.31	21.75 ± 0.94	33.60 ± 1.457
6.	**2c**	2.59 ± 0.11	18.74 ± 1.27	27.70 ± 4.390
7.	**3a**	25.31 ± 2.65	>100	>100
8.	**3b**	>100	>100	>100
9.	**3c**	46.07 ± 3.66	>100	>100
10.	**4a**	>100	>100	>100
11.	**4b**	22.87 ± 2.30	59.73 ± 4.58	66.99 ± 7.19
12.	**5a**	>100	>100	>100
13.	**5b**	41.44 ± 8.49	87.01 ± 5.08	86.19 ± 2.18
Eflornithine		23.14 ± 3.05		
Pentamidine		0.005 ± 0.00	4.34 ± 0.17	1.24 ± 0.11

All EC_50_ values were obtained using the Alamar Blue assay and are given as averages in µM (±SEM), of 3 independent evaluations.

**Table 2:** Chemical structures of tested synthetic chalcone derivatives [20].

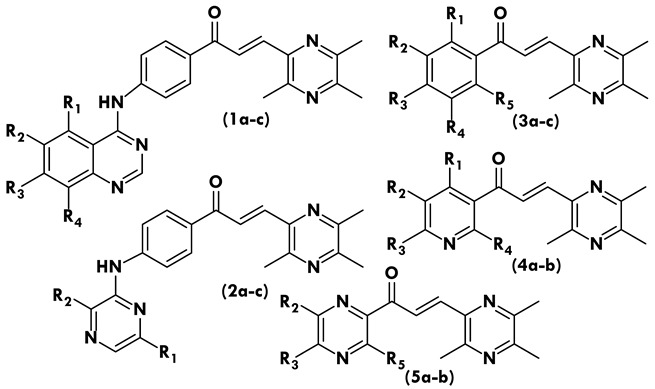					
**Compound**	**R_1_**	**R_2_**	**R_3_**	**R_4_**	**R_5_**
**1a**	**-**	**-**	**-**	**-**	**-**
**1b**	**-**	Cl	-	**-**	**-**
**1c**	**-**	OCH_3_	OCH_3_	OCH_3_	-
**2a**	**-**	**-**	**-**	**-**	**-**
**2b**	CH_3_	-	**-**	**-**	**-**
**2c**	-	CH_3_	**-**	**-**	**-**
**3a**	**-**	**-**	CH_3_	**-**	**-**
**3b**	-	-	CH_3_	**-**	CH_3_
**3c**	-	CH_3_	CH_3_	**-**	CH_3_
**4a**	**-**	**-**	OCH_3_	**-**	**-**
**4b**	**-**	**-**	OCH_3_	OCH_3_	**-**
**5a**	**-**	**-**	**-**	**-**	**-**
**5b**	**-**	**-**	**-**	**-**	CH_3_

## Data Availability

All data has been provided in this article.
